# UAV Path Planning for Reconnaissance and Look-Ahead Coverage Support for Mobile Ground Vehicles

**DOI:** 10.3390/s21134595

**Published:** 2021-07-05

**Authors:** Herath M. P. C. Jayaweera, Samer Hanoun

**Affiliations:** Institute for Intelligent System Research and Innovation, Deakin University, Melbourne, VIC 3125, Australia; samer.hanoun@deakin.edu.au

**Keywords:** UAV path planning, artificial potential field, reconnaissance and look-ahead coverage

## Abstract

Path planning of unmanned aerial vehicles (UAVs) for reconnaissance and look-ahead coverage support for mobile ground vehicles (MGVs) is a challenging task due to many unknowns being imposed by the MGVs’ variable velocity profiles, change in heading, and structural differences between the ground and air environments. Few path planning techniques have been reported in the literature for multirotor UAVs that autonomously follow and support MGVs in reconnaissance missions. These techniques formulate the path planning problem as a tracking problem utilizing gimbal sensors to overcome the coverage and reconnaissance complexities. Despite their lack of considering additional objectives such as reconnaissance coverage and dynamic environments, they retain several drawbacks, including high computational requirements, hardware dependency, and low performance when the MGV has varying velocities. In this study, a novel 3D path planning technique for multirotor UAVs is presented, the enhanced dynamic artificial potential field (ED-APF), where path planning is formulated as both a follow and cover problem with nongimbal sensors. The proposed technique adopts a vertical sinusoidal path for the UAV that adapts relative to the MGV’s position and velocity, guided by the MGV’s heading for reconnaissance and exploration of areas and routes ahead beyond the MGV sensors’ range, thus extending the MGV’s reconnaissance capabilities. The amplitude and frequency of the sinusoidal path are determined to maximize the required look-ahead visual coverage quality in terms of pixel density and quantity pertaining to the area covered. The ED-APF was tested and validated against the general artificial potential field techniques for various simulation scenarios using Robot Operating System (ROS) and Gazebo-supported PX4-SITL. It demonstrated superior performance and showed its suitability for reconnaissance and look-ahead support to MGVs in dynamic and obstacle-populated environments.

## 1. Introduction

Unmanned aerial vehicles (UAVs) are aircrafts that fly without a pilot onboard. They have been extensively deployed for assisting in military missions such as reconnaissance [1], surveillance [2], and combat operations [3]. Recently, UAVs have been utilized in other sectors supporting different commercial [4,5,6,7,8], environmental [9], and leisure [4] applications. Examples include monitoring of construction sites [10], inspection of civil infrastructures [10], surveying powerlines [7], mapping gas pipelines [11], counting agriculture livestock [5], assisting with forest fires [9], and mostly in cinema and photography for professional and leisure purposes [4]. In these applications, the UAV is usually equipped with appropriate thermal and visual sensors to effectively capture live views and photos for the objects and areas of interest, which are either stored onboard or relayed to a base station for further online and offline analysis.

Classification of UAVs can be based generally on their wing configuration as fixed-wing, rotary-wing, and hybrid. Unlike fixed-wing UAVs, rotary-wing UAVs are less expensive and provide great advantages for easy takeoff, landing, and hovering; however, they have limited endurance and speed compared to fixed-wing UAVs [6]. They use rotating propellers to generate thrust and control their lift, similar to helicopters. Multirotor UAVs are the most popular type of rotary-wing UAVs with two or more propellers. They are widely used in many civilian commercial applications for monitoring and inspection activities as they have great abilities to perform quick turns, maneuvers, and maintain velocity. The ease of their path planning having less constraints compared to fixed-wing UAVs and the ability to easily and quickly takeoff and land specially on moving vehicles make them worthy for following and supporting mobile ground vehicles (MGVs) in immediate reconnaissance and look-ahead missions for providing extended aerial sensing and coverage beyond the MGVs’ capabilities [12].

To date, UAVs have shown to be efficient and effective at following MGVs for tracking purposes [7,8,11]; however, delivering immediate and instant reconnaissance and look-ahead coverage when needed is another feasible, effective, and vital application for multirotor UAVs that has not been explored yet. In scenarios such as a vehicle moving in a dynamic and unknown terrain, a fire truck approaching a fire area, and a law enforcement vehicle exploring unattended areas, a multirotor UAV can be launched to autonomously follow the moving vehicle, at a standoff distance, to collect and relay in real-time additional aerial mapping information and visual coverage of the vehicle’s routes and areas ahead, thus enhancing the MGVs’ situational awareness level beyond their onboard sensors’ coverage abilities.

Path planning is a critical component to achieve the reconnaissance and look-ahead support for MGVs as the planned path should be safe, flyable, feasible, accurate, effective, and easy to compute. In a real-world unstructured and dynamic environment, the UAV path planning technique should allow avoiding both static and dynamic obstacles while considering the MGV’s change in velocity, heading, and altitude. In such a case, the path planning technique needs to satisfy three objectives, namely: (a) follow the MGV reliably; (b) provide a sufficient amount of coverage; and (c) provide the necessary coverage quality for the MGV’s route ahead. Existing path planning techniques for following MGVs have been mainly developed for fixed-wing UAVs [8,12,13]. They heavily rely on using gimbal cameras to aim at specific areas for coverage and tracking purposes [14,15,16], therefore, relaxing the complexities imposed by the path planning technique. Additionally, gimbal cameras are expensive, prone to failure, and consume more power than nongimbal cameras, thus affecting the UAV’s endurance and flight time. Moreover, these attempts have simplified the path planning problem by considering only two-dimensional path planning without obstacle avoidance and static environments, which do not fit real-world applications, while overlooking the pixel density and maximum coverage tradeoffs.

In this study, a novel online 3D path planning technique was proposed for a multirotor UAV equipped with a nongimbal camera to support an MGV with reconnaissance and surveillance of its route ahead, also called look-ahead coverage. The proposed path planning technique was based on the general artificial potential field (APF) method and, hence, is referred to as the enhanced dynamic artificial potential field (ED-APF). The proposed technique simultaneously solves both the follow and coverage problems. The ED-APF extends the dynamic artificial potential field (D-APF) [17], which enables a UAV to follow an MGV at a constant relative altitude and dynamically adapt to its variable velocity, by adding a vertical force component to the UAV to follow the MGV in a vertical sinusoidal path with a constant wavelength while alternating between broad and precise coverage. The altitude change enables the UAV to extend the MGV look-ahead and exploration area, thus extending its reconnaissance abilities. Besides, the additional relative velocity component of the force function improves the path planning performance in dynamic obstacle-populated environments and adapts the UAV relative to the MGV’s motion profile including the path and velocity.

The remainder of the paper is structured as follows. Section 2 presents the existing literature on path planning techniques for MGV reconnaissance and look-ahead support. Section 3 highlights the artificial potential field (APF) techniques, while Section 4 demonstrates the details of the proposed ED-APF technique. Section 5 details the setup of the simulation experiments with the results and discussion. Finally, Section 6 presents the conclusion and future work.

## 2. Related Work

Path planning is a vital process that enhances the deployment ability of UAVs for autonomous missions. In an unknown environment, the task of path planning is not only to navigate in a collision-free path, but also to accomplish the mission objectives.

The existing path planning techniques for unmanned vehicle navigation can be classified into two subgroups based on the type of information used for the path planning: global (offline) and local (online) path planning. Pure pursuit [18], A* [19], and mathematical model-based algorithms [20] are examples of global path planning that requires a priori knowledge about the navigation environment. This method creates a map using the samples available to determine the optimal path. The inability to deal with unknown mobile obstacles is the main drawback of global path planning. Therefore, global path planning is not a feasible solution, on its own, for autonomous UAVs in unknown environments.

Local path planning such as rapidly exploring random tree (RRT) [21], the artificial neural network [22], the genetic algorithm [23], and fuzzy logic [24] requires real-time environmental information to process the navigation. Therefore, these methods can be adopted for UAV navigation in unknown environments. For example, the RRT-based methods in [21,25] were capable of collision-free navigation planning, but had poor performances in optimization, lacked a replanning procedure, and had a computational complexity that increased exponentially for large-area path planning. However, in addition to point-to-point navigation, the RRT can be adopted for motion planning with dynamic awareness [26] and the exploration of unknown and cluttered environments [27]; the solution is not always the optimal due to the lack of adequate local information, but it is a feasible one. Besides, these solutions are unable to deal with dynamic obstacles. However, the work in [28] presented a chance-constrained motion planning that was capable of dynamic obstacle avoidance, but the algorithm was complex and could not directly be used for waypoint navigation.

In real-world applications, path planning is formulated as an optimization problem as the environment is stochastic and dynamic and cannot fully be predicted in advance. In order to overcome these problems, a technique called model predictive control (MCP) is adopted for path planning of unmanned vehicles [29,30,31]. These path planning techniques solve a sequence of optimization problems in a recursive manner and account for the change of the environment conditions during the planning process.

Current UAV path planning techniques for autonomously following MGVs for reconnaissance and look-ahead support have been proposed mainly for fixed-wing UAVs. They can be classified into two groups, loitering and following, based on the UAV’s minimum air velocity and the MGV’s ground velocity. It is well known that fixed-wing UAVs must maintain a minimum air velocity to remain airborne as they generate the required lift in accordance with their air velocity. When the MGV’s velocity is less than the UAV’s minimum air velocity, the path planning has to adopt a loitering mode by flying around the MGV in a two-dimensional sinusoidal [12], circular [32], or square [33] pattern path to compensate for the difference in velocities. The main drawback of this method is its inability to continue the shape of the path in the presence of obstacles and sudden changes in the MGV’s velocity and heading. Loitering techniques depend on gimbal sensors, to minimize the loss in coverage while loitering; however, gimbal sensors are expensive and exhibit a high level of power consumption. When the MGV’s velocity is between the minimum and maximum UAV’s air velocities, the path planning switches to following the movement of the MGV with the UAV either trailing or leading the MGV’s position. Although fixed-wing UAVs have high endurance, they are unable to perform quick turns and suddenly change their air velocity and heading. Therefore, fixed-wing UAV path planning techniques fail to follow the MGV when it changes its velocity and heading due to the nature of the ground environment being unstructured, dynamic, and obstacle-populated.

In contrast, multirotor UAVs can quickly accommodate by making quick turns, hovering, and changing their velocities swiftly, which makes them more suitable for following MGVs and providing reconnaissance and look-ahead support in dynamic environments and at low altitudes. Several path planning techniques for multirotor UAVs following MGVs have been reported in the literature. A common approach adopts the proportional–integral–derivative (PID) function [34] and the proportional–derivative (PD) function [17]; this approach works well with adequate position and velocity information. However, providing sufficient and accurate position and velocity information at an adequate frequency can be challenging in most environments, especially when there is no direct line-of-sight between the MGV and the UAV due to obstacles. Another approach integrates fuzzy logic with the PD or PID controller [35]. However, this integration increases the overshoot and risk of colliding with obstacles in a dynamic environment as the fuzzy controller usually requires more computational time to produce the UAV flight path. An analytical expression for fuzzy PI-based path planning consisting of 49 rules to control the pitch and rolling angles to follow an MGV has been demonstrated in simulations and experiments [36]. This technique requires a gimbal camera that collects on-time information about the MGVs environment because the yaw angle is not controlled.

Another interesting approach, an APF-based path planning technique for following MGVs, was presented for indoor applications [37]. This method uses two forces for guidance, namely an attractive force by which the UAV is drawn towards the MGV and a repulsive force to repel it from obstacles. This ensures that the UAV follows the MGV and avoids obstacles simultaneously. Although this method provides waypoints that can easily be implemented in an existing autopilot such as PX4-autopilot [38] or ArduPilot [39], symmetric obstacles have proven to be challenging to handle. An enhanced version of the general APFs, the dynamic-APF (D-APF) [40], accommodates an approach to change the UAV’s altitude in the presence of obstacles to ensure collision avoidance when the UAV is following the MGV. The D-APF repulsive force that controls and changes the UAV’s altitude when obstacles are in the planned path of the UAV is active along the vertical direction. The repulsive force due to the *n*th obstacle is defined as follows:

(1) $$\begin{array}{c} {}^{n}F_{r}\left(Z\right)=\frac{p_{o,a}\left(z\right)}{|p_{o,a}\left(z\right)|}\left[c_{1}\;e^{-k_{1}{|}^{n}p_{o,a}\;\cos\theta |}+c_{2}\;e^{-k_{2}{|}^{n}v_{o,a}|}\right];\;c_{1}=0\;if{\;}^{n}p_{o,a}\geq 0,\\ \;c_{2}=0\;if{\;}^{n}v_{o,a}<0\;and{\;}^{n}p_{o,a}\geq h \end{array}$$

where $c_{1}$ and $c_{2}$ are the gain factors and the summation $c_{1}+c_{2}$ gives the maximum magnitude of the repulsive force on the horizontal plane. $k_{1}$ and $k_{2}$ are constants that help determine the minimum required relative displacement and velocity, respectively, to achieve the maximum magnitude of the repulsive force due to the nth obstacle. θ is the angle between the UAV’s heading and relative displacement between the *n*th obstacle and the UAV in the horizontal plane.

Even though the main task of most MGV following techniques is to access adequate information about the MGV’s surroundings, all of these path planning techniques were solely developed for following MGVs without providing any kind of reconnaissance and look-ahead support. Additionally, they neither consider the quality and quantity of the sensed information by the onboard camera, nor the quality and quantity tradeoffs and their correlation with the planned path.

## 3. General APFs for Following MGVs

Existing APF-based path planning techniques are commonly used to follow an MGV at a constant relative altitude in an obstacle-free and open environment. The APF is based on generating a virtual potential field where the UAV is regarded as a freely moving charge particle pulled towards the target by an attractive force and repelled from obstacles by a repulsive force. The attractive force is a function of the relative displacement and relative velocity between the UAV and the MGV, whereas the repulsive force is a function of the relative displacement and relative velocity between the UAV and the obstacles [37]. In general, there are two types of potential functions: general APF (G-APF) and general exponential APF (GE-APF). The general APFs lack features that allow a UAV to follow an MGV at a standoff distance and the control of the UAV altitude. Therefore, they were modified here to support these features and to allow for the comparison with the proposed technique.

### 3.1. General APF

The G-APF force function is a linear expression that is commonly used for the path planning of unmanned aerial vehicles [37]. The attractive force $F_{a}$ of the G-APF is modified as follows:

(2) $$F_{a}=F_{a}\left(p\right)+F_{a}\left(v\right)$$

(3) $$F_{a}\left(p\right)=c_{3}\left(p_{g,a}-s\right)+\left(c_{4}\;\frac{p_{g,a}}{|p_{g,a}|}\right);\;c_{3}=0\;if\;p_{g,a}>p_{d},\;else\;c_{4}=0$$

(4) $$F_{a}\left(v\right)=c_{5}\;v_{g,a}+\left(c_{6}\;\frac{v_{g,a}}{|v_{g,a}|}\right);\;c_{5}=0\;if\;v_{g,a}>v_{d},\;else\;c_{6}=0$$

where $F_{a}\left(p\right)$ and $F_{a}\left(v\right)$ are the attractive forces due to the relative displacement $p_{g,a}$ and relative velocity $v_{g,a}$, respectively. The variable constants $c_{3},\;c_{4},\;c_{5},$ and $c_{6}$ determine the linear and constant ranges of the force function, where $p_{d}$ and $v_{d}$ are the linear ranges of the displacement and velocity, respectively. The standoff displacement is represented by *s*, where s=(0,0,d) represents a zero standoff distance in the horizontal direction and *d* the standoff distance in the vertical direction (i.e., in altitude).

Similarly, the repulsive force $\left(F_{r}\right)$ of the G-APF is modified as follows:

(5) $$F_{r}=\sum \;{\{}^{n}F_{r}\left(p\right){+}^{n}F_{r}\left(v\right)\}$$

(6) $${}^{n}F_{r}\left(p\right)=\frac{-c_{r1}{\;}^{n}p_{o,a}}{{|}^{n}p_{o,a}-p_{e}{|}^{3}};\;c_{r1}=0\;if{\;}^{n}p_{o,a}>p_{m},\;else\;c_{r1}>0$$

(7) $${}^{n}F_{r}\left(v\right)=-c_{r2}{\;}^{n}v_{o,a};\;c_{r2}=0\;if{\;}^{n}v_{o,a}>0,\;else\;c_{r2}>0$$

where ${}^{n}F_{r}\left(p\right)$ and ${}^{n}F_{r}\left(v\right)$ are the repulsive forces due to the relative position and relative velocity, respectively, of the UAV and *n*th obstacle. $c_{r1}$ and $c_{r2}$ are variable scalar factors with magnitudes dependent on the relative displacement and relative velocity, respectively, of the obstacle and UAV. The parabolic range of the displacement is denoted as $p_{m}$. The total force *F* acting on the UAV is given by the vector summation of the resultant attractive and repulsive forces:

(8) $$F=F_{a}+F_{r}$$

### 3.2. General Exponential APF

The GE-APF function is an exponential potential field expression that is commonly used for the path planning of unmanned ground vehicles [41]. However, a 3D version of the GE-APF can be used to allow a UAV to follow an MGV. The typical GE-APF is modified here by introducing a standoff distance to the potential field expression. The attractive and repulsive forces of the modified GE-APF can be expressed as follows:

(9) $$F_{a}=\frac{p_{g,a}}{|p_{g,a}|}\;\left[c_{7}\;\left(1-e^{-k_{3}\left(p_{g,a}^{2}-s^{2}\right)}\right)+c_{8}\;\left(1-e^{-k_{4}\;v_{g,a}^{2}}\right)\right]$$

(10) $${}^{n}F_{r}=\frac{p_{o,a}}{|p_{o,a}|}\;\left[c_{9}\;\left(e^{-k_{5}{\;}^{n}p_{o,a}^{2}}\right)+c_{10}\;\left(1-e^{-k_{6}{\;}^{n}v_{o,a}^{2}}\right)\right];\;k_{6}=0\;if{\;}^{n}v_{o,a}>0$$

where $c_{7},\;c_{8},\;c_{9},$ and $c_{10}$ are scalar gain factors and the summations $c_{7}+c_{8}$ and $c_{9}+c_{10}$ give the maximum attractive and repulsive forces, respectively. $k_{3},\;k_{4},\;k_{5},$ and $k_{6}$ are positive constants that control the required minimum displacement and maximum velocity to achieve the maximum attractive and repulsive forces.

## 4. Enhanced Dynamic APF

The objective of any APF technique is to obtain a mathematical model that generates force fields without sharp changes in the force field continuum. Abrupt changes in the force field continuum can create sharp changes in the power demand for the UAV motors, which can produce motion limitations and cause the potential failure of mechanical components. Having a smooth force field continuum is imperative for optimal field performance when the APF mathematical model is adopted for planning the UAV path. In simpler terms, the model needs to generate smooth transitions in the force fields when the UAV is approaching obstacles and following the MGV. Mathematically, sinusoidal paths provide smooth transitions for functional outputs compared to the input variables. This makes sinusoidal functions very useful for the development of path planning models.

The proposed ED-APF was inspired by and extends and enhances the techniques presented in [36,37,40]. The proposed ED-APF generates velocity waypoints by simultaneously considering the constraints of following the MGV and the visual coverage quality of the reconnaissance routes and areas. This is achieved by adopting the concept of the D-APF [40] for path planning and enhancing the attractive force for following the MGV along a vertical sinusoidal path. The ED-APF’s attractive force controls and adapts to the frequency of the vertical sinusoidal path based on the MGV’s velocity, the camera’s field-of-view (FoV), the average altitude, and the camera fixed gimbal angle. The average altitude is a function of the standoff distance between the UAV and the MGV and the tradeoff between the coverage and pixel density.

The proposed ED-APF can be adopted by any UAV having a flight controller with autopilot features, basic sensors onboard the UAV for measuring distance and position, an onboard nongimbal camera that is fixed at a specific position, and a companion computer, as well as an MGV that can relay its positional information to the UAV and receives the captured visual information by the UAV’s onboard camera. The ED-APF generates the UAV’s velocity waypoints by using two types of forces: an attractive force towards the MGV for following it and a repulsive force from obstacles for collision-free navigation.

### 4.1. ED-APF Attractive Force

The resultant force of the two attractive forces along the horizontal plane $F_{a}\left(xy\right)$ and vertical axis $F_{a}\left(z\right)$ is adopted to generate the required velocity waypoints for following the MGV with a variable velocity in a dynamic environment. The attractive force $F_{a}\left(xy\right)$ in the horizontal direction is a modified version based on the D-APF attractive force [40], while $F_{a}\left(z\right)$ was proposed and defined to control the vertical sinusoidal path based on the MGV’s velocity $\left(v_{g}\right)$, the onboard camera’s FoV on the horizontal plane (HFoV=α) and the vertical plane (VFoV=β), the minimum relative altitude of the UAV $\left(h_{min}\right)$, and the camera-fixed gimbal angle (θ), as follows:

(11) $$F_{a}\left(xy\right)=\frac{p_{g,a}}{|p_{g,a}|}\;\left[c_{11}\;\left(1-e^{-k_{7}\left|p_{g,a}-s\right|}\right)+c_{12}\;\left(1-e^{-k_{8}\;\left|v_{g,a}\right|}\right)\right]$$

(12) $$F_{a}\left(z\right)=\frac{p_{g,a}\left(z\right)}{|p_{g,a}\left(z\right)|}\;\left[c_{13}\;\left(1-e^{-k_{9}\left|p_{g,a}\left(z\right)-h\right|}\right)+A\;\sin\left(\frac{\pi \;p_{g,a}\left(xy\right)}{L_{1}+h_{min}\;\tan\left(\theta -\frac{\beta }{2}\right)}\right)\right]$$

(13) $$L_{1}=h_{min}\;\left\{\tan\left(\frac{\beta }{2}+\theta \right)+\tan\left(\frac{\beta }{2}-\theta \right)\right\}$$

(14) $$\theta =\tan^{-1}\left(\frac{L_{1}+h_{min}\;\tan\left(\theta -\frac{\beta }{2}\right)}{h_{min}}+\frac{\beta }{2}\right)$$

(15) $$A=\frac{1}{2}\left(\sqrt{\frac{n}{\rho_{min}\;\phi }}-h_{min}\right)$$

(16) $$\phi =\left\{\tan\left(\frac{\beta }{2}+\theta \right)+\tan\left(\frac{\beta }{2}-\theta \right)\right\}\;\left[\frac{\tan(\alpha /2)}{\cos(\theta -\beta /2)}+\frac{\tan(\alpha /2)}{\cos(\theta +\beta /2)}\right]$$

where $c_{11}$ and $c_{12}$ are the gain factors of the attractive force and the summation $c_{11}+c_{12}$ gives the maximum magnitude of the attractive force. The constants $k_{7}$ and $k_{8}$ control the minimum required displacement and velocity, respectively, to achieve the maximum magnitude of the force.

**Figure 1:**
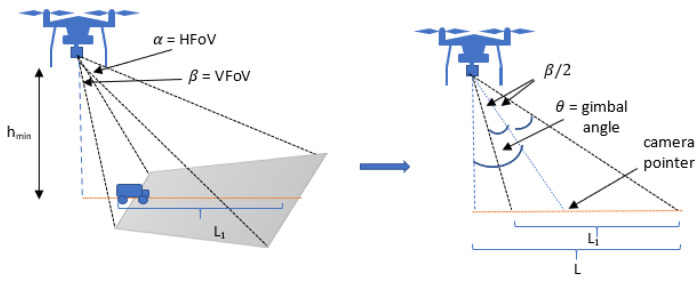
A side view illustration of the UAV’s onboard nongimbal camera coverage at the UAV’s minimum flying altitude.

As shown in Figure 1, *L* represents the wavelength, and $L_{1}$ is the look-ahead distance of the UAV at its minimum altitude and is calculated by substituting the VFoV, gimbal angle, and $h_{min}$ in (13).

In order to collect the look-ahead aerial information from the UAV’s attached fixed camera, the UAV needs to synchronize its heading with the MGV’s heading. Therefore, the relative heading between the UAV and the MGV $\left(\gamma_{g,a}\right)$ was used to introduce a novel attractive force for the heading $\left(F_{\gamma }\right)$ as follows:

(17) $$F_{\gamma }=\frac{\gamma_{g,a}}{|\gamma_{g,a}|}\;g_{1}\;\left(1-e^{-g_{2}\;\left|\gamma_{g,a}\right|}\right)$$

where $g_{1}$ and $g_{2}$ are constants where the maximum attractive force for the heading is given by $g_{1}$. The positive constant $g_{1}$ is used to control the minimum relative heading required to achieve the maximum attractive force for the heading.

### 4.2. ED-APF Repulsive Force

In real-world applications and for most environments, ground and air obstacles differ in shape and location, as they might not be the same for the MGV and the UAV and mostly require different avoidance strategies. For example, in a forest environment, the shapes of trees are not unique at the bottom and top, while in an urban environment, man-made structures, such as bridges, exist at one level, while not at the other. The general APFs’ path planning techniques try to avoid obstacles by maneuvering around them in a two-dimensional approach; however, they fail to produce a collision-free path when facing symmetric obstacles. They also suffer from local minima and create unnecessarily long paths when obstacles are nearby. Other technique such as the D-APF [40] plans collision-free paths by changing the UAV’s altitude in the vertical direction; however, it does not compensate and unable to handle dynamic obstacles moving towards the UAV. The ED-APF’s resultant repulsive force is proposed to overcome these drawbacks as it consists of two subrepulsive forces to produce a collision-free path while continuously following the MGV. The first subrepulsive force acts in the vertical direction to enable the UAV to change its altitude to prevent any collision and is a function of the relative displacement $\left(p_{o,a}\right)$ and relative velocity $\left(v_{o,a}\right)$ of the UAV and the obstacles:

(18) $$\begin{array}{c} {}^{n}F_{r1}\left(z\right)=\frac{p_{o,a}\left(z\right)}{|p_{o,a}\left(z\right)|}\left[c_{1}{}_{4}\;e^{-k_{10}{|}^{n}p_{o,a}\;cos\delta |}+c_{15}\;e^{-k_{11}{|}^{n}v_{o,a}|-k_{12}{|}^{n}p_{o,a}|}\right];\;\\ c_{14}=0\;if{\;}^{n}p_{o,a}\geq 0,\;c_{15}=0\;if{\;}^{n}v_{o,a}<0\;and{\;}^{n}p_{o,a}\geq h \end{array}$$

$c_{14}$ and $c_{15}$ are gain factors, where the summation $c_{14}+c_{15}$ gives the maximum magnitude of the repulsive force in the vertical plane. $K_{10}$ and $k_{11}$ are constants that help determine the minimum relative displacement and velocity, respectively, to achieve the maximum magnitude of the repulsive force due to the *n*th obstacle. δ is the instantaneous angle between $V_{g,a}\left(x,y\right)$ and $p_{o,a}\left(xy\right)$.

The second subrepulsive force controls the horizontal motion of the UAV to avoid collisions when the first subrepulsive force is not sufficient. The second subrepulsive force is the resultant force of two components, namely the force due to the relative position ${}^{n}F_{rp}\left(xy\right)$ and the force due to the relative velocity ${}^{n}F_{rv}\left(xy\right)$:

(19) $$\begin{array}{c} {}^{n}F_{rp}\left(xy\right)=\left\{\begin{array}{c} F_{a}\left(xy\right)\;\left(e^{-k_{12}{|}^{n}p_{o,a}\left(xy\right)-a_{1}|}\right);{\;}^{n}p_{o,a}\left(xy\right)\;\cos\delta \leq a_{1}\\ \frac{p_{o,a}}{|p_{o,a}|}\;c_{16}\;e^{-k_{13}\;{|}^{n}p_{o,a}|};{\;}^{n}p_{o,a}\left(xy\right)\leq a_{2}\\ 0;{\;}^{n}p_{o,a}\left(xy\right)>a_{2} \end{array}\right. \end{array}$$

(20) $$\begin{array}{c} {}^{n}F_{rv}\left(xy\right)=\left\{\begin{array}{c} \frac{p_{o,a}}{|p_{o,a}|}\;c_{17}\;e^{-k_{14}\;{|}^{n}v_{o,a}|};{\;}^{n}v_{o,a}<0\;and{\;}^{n}p_{o,a}\leq a_{2}\\ 0;{\;}^{n}v_{o,a}\leq \;and{\;}^{n}p_{o,a}>a_{2} \end{array}\right. \end{array}$$

(21) $${}^{n}F_{r2}\left(z\right)={\;}^{n}F_{rp}\left(xy\right)+{\;}^{n}F_{rv}\left(xy\right)$$

where $c_{16}$ and $c_{17}$ are gain factors, where the summation $c_{16}+c_{17}$ gives the maximum magnitude of the repulsive force in the horizontal plane when $p_{o,a}\leq a_{2}$. $k_{12},\;k_{13}$, and $k_{15}$ are constants that help to determine the minimum relative displacement to achieve the maximum magnitude of the horizontal repulsive force. $k_{14}$ is also a constant that helps to determine the minimum relative velocity to achieve the maximum magnitude of the horizontal repulsive force.

### 4.3. Waypoint Generation

For a UAV with a mass *m* and a velocity $v_{t}$, the next velocity waypoint $v_{t+\delta t}$ can be expressed as follows:

(22) $$v_{t+\delta t}=\left(1+\frac{1}{f}\right)\;v_{t}\;+\frac{\sum F}{m\;f}$$

where *f* is the waypoints’ transfer frequency to the autopilot and ∑F is the ED-APF’s resultant force.

**Figure 2:**
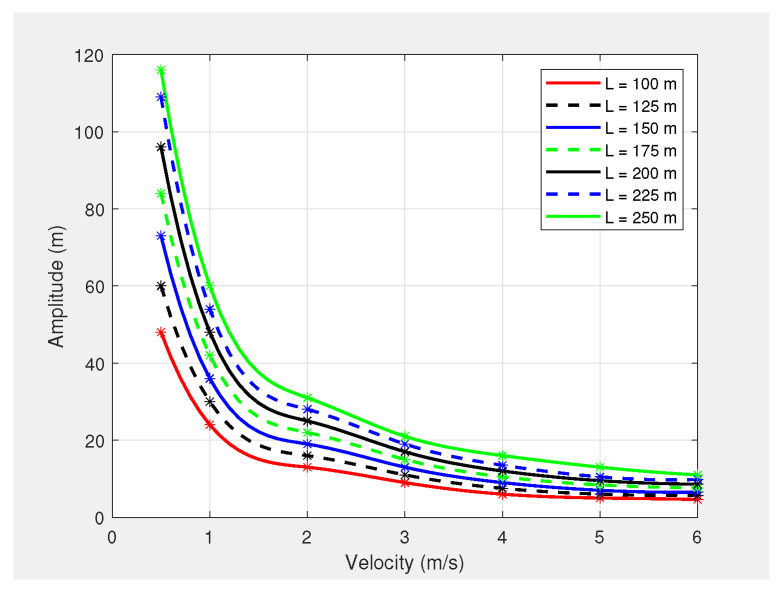
PX4-autopilot’s supported maximum amplitude vs. velocity for different wavelengths.

Although the amplitude of the UAV’s planned path from (15) is independent of the UAV’s horizontal velocity, the PID controller of the PX4-autopilot limits the amplitude based on the UAV’s horizontal velocity. This is due to the inability of the PX4-autopilot to perform sharp turns at either a positive or negative peak of the sinusoidal path when the UAV is moving at a high speed in the horizontal direction. Figure 2 shows a graph of the PX4-autopilot’s supported maximum amplitude versus velocity for different wavelengths. Therefore, (15) was reformulated to enable the generation of the appropriate waypoints for the PX4-autopilot’s offboard mode, as follows:

(23) $$A=minimum\left(\frac{1}{2}\left(\frac{n}{\sqrt{\rho_{min}\;\phi }}-h_{min}\right),\;max\left[\gamma \left(v\right)\right]\right)$$

(24) $$\begin{array}{c} \gamma \left(v\right)=\left\{\begin{array}{c} 0.0602v^{6}-1.3419v^{5}+11.996v^{4}-54.799v^{3}+135.05v^{2}-174.68v\\ +107.72;\;100\leq L\\ 0.0787v^{6}-1.7308v^{5}+15.279v^{4}-69.077v^{3}+169.08v^{2}-218.18v\\ +134.55;\;125\leq L\\ 0.1032v^{6}-2.2466v^{5}+19.624v^{4}-87.839v^{3}+213.05v^{2}-272.51v\\ +165.81;\;150\leq L\\ 0.112v^{6}-2.447v^{5}+21.462v^{4}-96.493v^{3}+235.46v^{2}-304.05v\\ +187.95;\;175\leq L\\ 0.1296v^{6}-2.821v^{5}+24.649v^{4}-110.49v^{3}+269.07v^{2}-347.27v\\ +214.72;\;200\leq L\\ 0.1546v^{6}-3.3439v^{5}+29.037v^{4}-129.37v^{3}+313.22v^{2}-401.71v\\ +246.01;\;225\leq L\\ 0.1525v^{6}-3.2785v^{5}+28.279v^{4}-125.42v^{3}+304.16v^{2}-395.73v\\ +251.84;\;250\leq L \end{array}\right. \end{array}$$

where γ(v) is the maximum amplitude that can be achieved through the PX4-autopilot, calculated from (24), where *v* is the horizontal velocity of the UAV.

To calculate the velocity waypoints, the path planner requires the MGV’s positional information and $\rho_{min}$ as inputs from the MGV’s and the UAV’s GPS and distance sensor data as inputs from the UAV’s onboard sensors. Besides, the UAV’s onboard camera’s HFoV and VFoV, $L_{1}$, the maximum sensing distance of the MGV’s onboard sensor, and the pre-defined variable constants are required for the velocity waypoints’ calculation. Three consecutive positional information were adopted for the MGV’s velocity and heading calculation. The camera fixed gimbal angle θ was calculated and set before starting where $L_{1},\;h_{min}$ and β values were used in (13) to find the adequate θ. The calculated θ, pre-defined $L_{1}$, β, and $h_{min}$, and the MGV required $\rho_{min}$ were used by the path planner to calculate *A* from (23). The PX4-autopilot accepts velocity waypoints in the X, Y, and Z directions; therefore, the resultant force acting on each direction given by (11), (12), and (21) was used in (22) to calculate the PX4-autopilot’s required velocity waypoints. The PX4-autopilot’s yaw angle waypoint from (17) was used to generate the required waypoints for the UAV’s yaw angle to maintain a synchronized heading with the MGV.

## 5. Simulation Experiments

The proposed ED-APF was implemented in the PX4-SITL (software in the loop) [38] supported by ROS [42] and Gazebo [43]. To evaluate its performance and suitability for MGVs’ reconnaissance and look-ahead coverage support, different simulation experiments were conducted where the MGV adopted constant and variable velocities while moving along straight and curved-type paths. The UAV’s planned path by the ED-APF, including position, velocity, and altitude, besides the look-ahead distance coverage in terms of quantity and quality, were evaluated. In each simulation experiment, the UAV and the MGV started their motions from (0,0,0) and (1,0,0), respectively. Both vehicles initiated their motions simultaneously; the UAV moved vertically, while the MGV moved horizontally, where the UAV started following the MGV once it reached an altitude of 15 m. In all simulation experiments, the minimum relative altitude for the UAV when following the MGV was set at 30 m to conform to the general aviation regulations requiring UAVs to keep a minimum flying distance of 30 m away from other entities such as vehicles and humans [44]. Furthermore, the ED-APF was examined for its ability to handle static and dynamic obstacles in the course of the UAV’s planned path such as symmetric, large, nearby, and flying obstacles. The details of the UAV and MGV models used in the simulation experiments and the ED-APF’s performance results are presented in the following sections.

### 5.1. Simulation Framework

**Figure 3:**
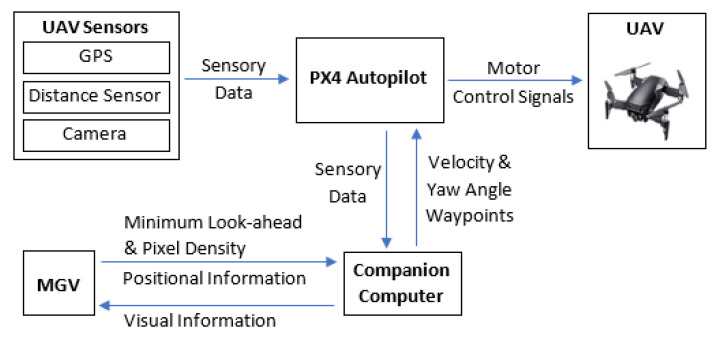
The simulation framework of the proposed ED-APF for MGV reconnaissance and look-ahead support.

The simulation framework of the proposed ED-APF is presented in Figure 3. The path planner receives input information for the MGV’s position, minimum look-ahead distance, and required pixel density range from the MGV at a frequency of 10 Hz and the UAV’s onboard sensory data from the PX4-autopilot through the MAVLink protocol. These sensory data included the UAV’s position, velocity, yaw angle, distance to obstacles and angle between the UAV’s heading and obstacles. The MGV’s position information was used to calculate its velocity and heading. The path planner generated the UAV’s velocity waypoints for following the MGV while avoiding obstacles and extending the reconnaissance of its route ahead.

### 5.2. UAV Model

The UAV model utilized in the simulation experiments was the PX4-SITL multirotor UAV “Iris” [45]. This multirotor UAV is equipped with a Global Positioning System (GPS) to determine its position, two distance sensors to support obstacle avoidance, and a micro air vehicle link (MAVLink) for communication with other entities such as other UAVs, base stations, and ground vehicles. The UAV has a flight controller aided by the PX4-autopilot and an inertial measurement unit (IMU) for providing its velocity and heading. The onboard autopilot accepts yaw angle, velocity, and position waypoints via the MAVLink. An application programming interface (API), MAVROS, runs on the companion computer to deliver the generated path planning waypoints as inputs to the UAV’s autopilot at a frequency of 2 Hz.

The onboard nongimbal camera mounted at the base of the UAV has an HFoV and VFoV of 84 and 61.9 degrees, a focal lens of f/2.8, and a resolution of 12.35 megapixels, as these are common properties for multirotor UAV’s cameras.

### 5.3. MGV Model

The MGV model adopted in the simulation experiments was the PX4-SITL unmanned ground vehicle “r1-rover” [45]. The ground vehicle model was modified to support it to achieve a maximum velocity of 6 m/s (i.e., 21.6 km/s) while being driven by velocity and yaw angle waypoints, thus enabling it to turn around its Z-axis without changing its X and Y positions. To support the MGV’s constant and variable velocity motion profiles, velocity waypoints with different constant velocities were used to set the MGV’s constant velocity at any value from 1 m/s to 5 m/s, while the yaw angle waypoints were utilized to control the MGV’s heading and direction. In the simulation experiments, the MGV followed a straight path of 1000 m (1 km) in length, while the curved path it followed was around 3000 m (3 km). Additionally, the visual sensor onboard the MGV was assumed to provide a look-ahead distance of 10 m.

### 5.4. Performance of the ED-APF for the MGV’s Constant Velocity Motion Profile

**Figure 4:**
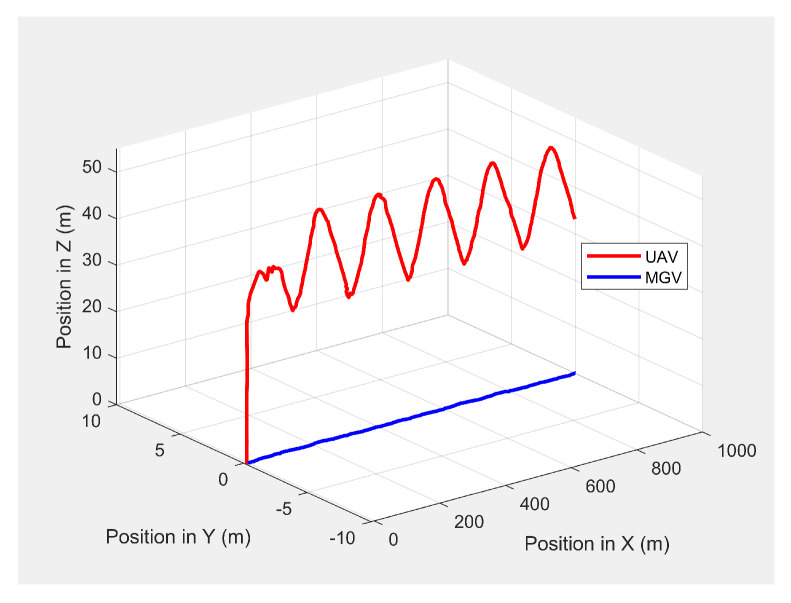
The UAV’s planned path in 3D when following the MGV while moving on a straight path.

**Figure 5:**
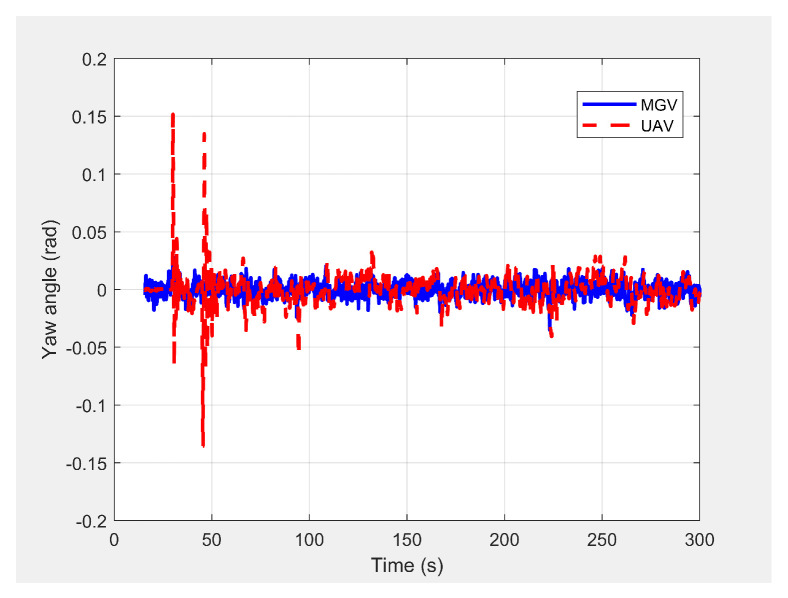
Variation in the UAV’s yaw angle when following the MGV while moving on a straight path.

Initially, the MGV’s constant velocity was set at 4 m/s along its 1000 m straight path to evaluate the UAV’s path generated by the ED-APF and look-ahead distance and coverage for different *L* values. The MGV’s requested visual quality was chosen as $\rho_{min}$ = 58 pixel/m${}^{2}$ to allow the UAV to make larger amplitudes and evaluate the effectiveness of the ED-APF algorithm. Figure 4 shows the 3D paths of the MGV and UAV when the MGV was moving at 4 m/s along the X-axis at a 175 m value of *L*. The UAV sinusoidal path had an amplitude of 10 m, a wavelength of 175 m, and a midline of 40 m. The UAV followed the MGV with an average position error of 0.14 m in the X-direction. Figure 5 shows the yaw angle variation where the UAV followed the MGV’s motion, maintaining a synchronized (i.e., the same) heading. The UAV’s heading had an average error of 0.0043 rad and a standard deviation of 0.0121 rad compared to the MGV’s heading. This was because the ED-APF’s attractive force in the horizontal direction and its heading were sensitive to small changes of relative displacement and relative heading even near the origin.

**Figure 6:**
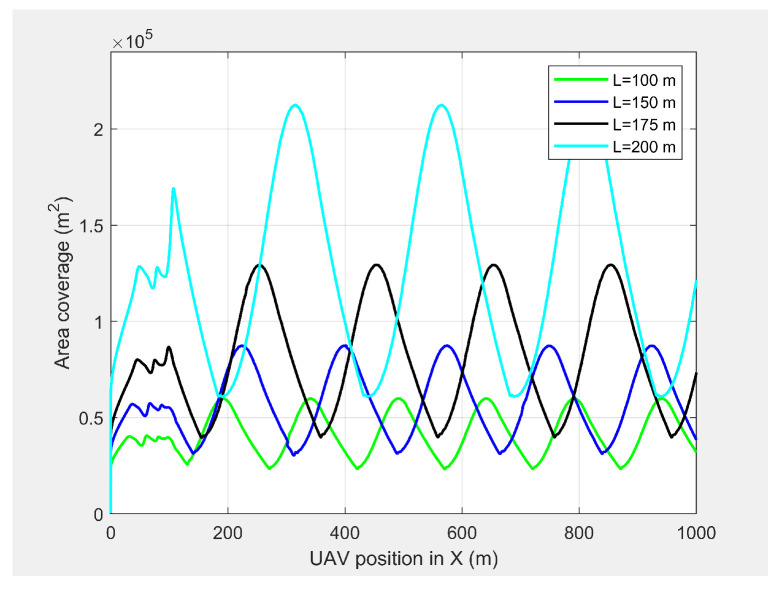
The UAV’s look-ahead coverage when following the MGV for different *L* values.

**Figure 7:**
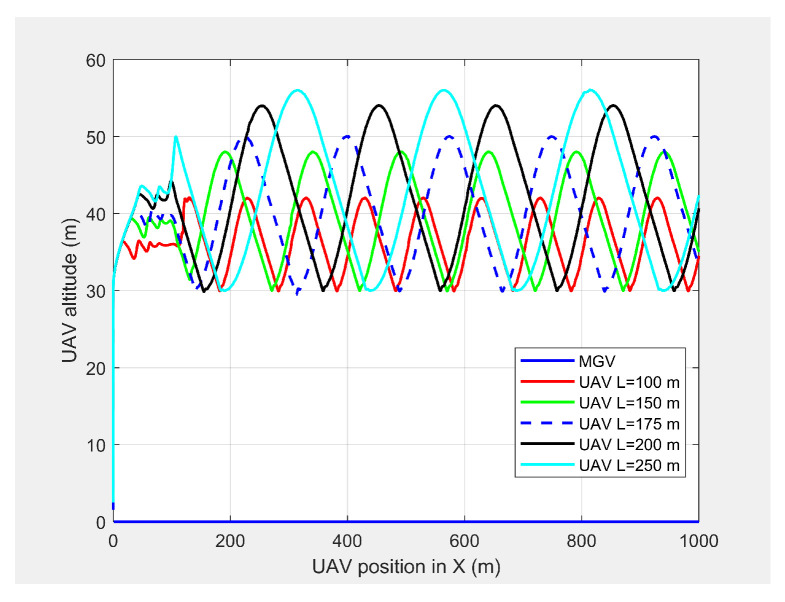
The UAV’s altitude when following the MGV for different *L* values.

Figure 6 and Figure 7 show the UAV look-ahead coverage and altitude variation, respectively. The look-ahead coverage was a function of the UAV’s altitude, so the maximum coverage was achieved at the crest of the sine wave, and the minimum coverage was achieved at the trough. Although, the UAV had the freedom to vary its altitude from 30 m to more than 50 m based on the pixel density, the PX4-autopilot was unable to complete the full sine wave with an altitude of 10 m for *L* < 175 m at 4 m/s.

Table 1 summarizes the look-ahead distance and coverage of the ED-APF for different $L_{1}$ and the corresponding θ. Increasing θ required increasing $L_{1}$ and *L*, which increased the look-ahead distance and coverage, as well as the minimum look-ahead distance of the MGV’s onboard sensors. Changing $L_{1}$ from 94 m to 190 m increased the maximum look-ahead distance by 160% and the maximum look-ahead coverage by 478%. In addition, changing *L* from 100 m to 200 m increased the minimum look-ahead distance by 98% and the minimum look-ahead coverage by 251%. This was because, for the selected MGV’s velocity, the UAV’s amplitude increased with *L*; therefore, the look-ahead distance and coverage increased with *L*.

**Figure 8:**
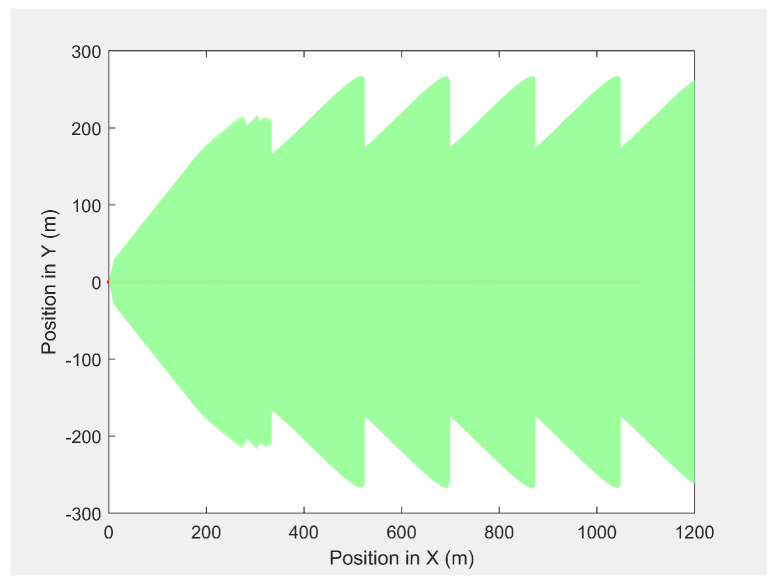
The UAV’s camera look-ahead coverage footprint along the MGV’s path.

Figure 8 presents the camera look-ahead coverage footprint along the MGV’s path for a distance of 1000 m, where the UAV had a maximum look-ahead distance of 275.6 m at an altitude of 50 m and a minimum look-ahead distance of 165.6 m at an altitude of 30 m. The maximum and minimum altitudes produced a maximum and a minimum look-ahead coverage of $8.73\times 10^{4}$ and $3.21\times 10^{4}\;m^{2}$, respectively. The average overlap distance was less than 0.1 m at a data transfer frequency of 40 Hz.

**Figure 9:**
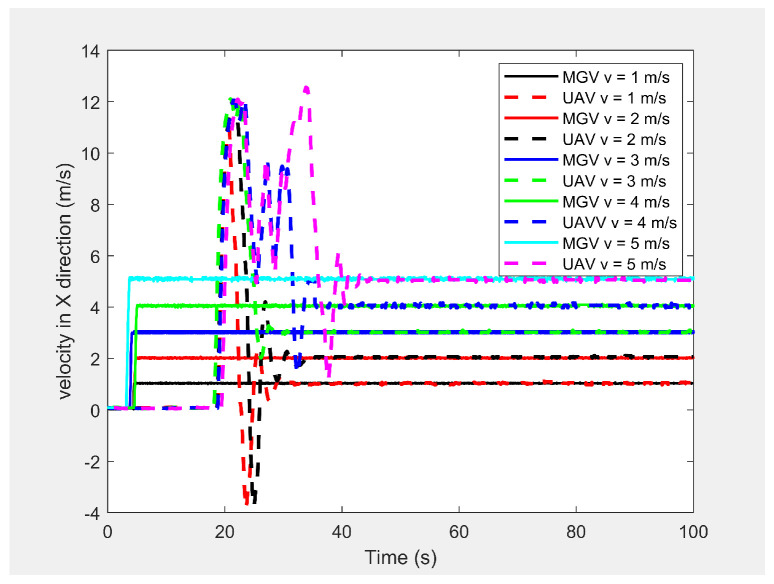
The UAV’s horizontal velocity for different constant velocities of the MGV.

**Figure 10:**
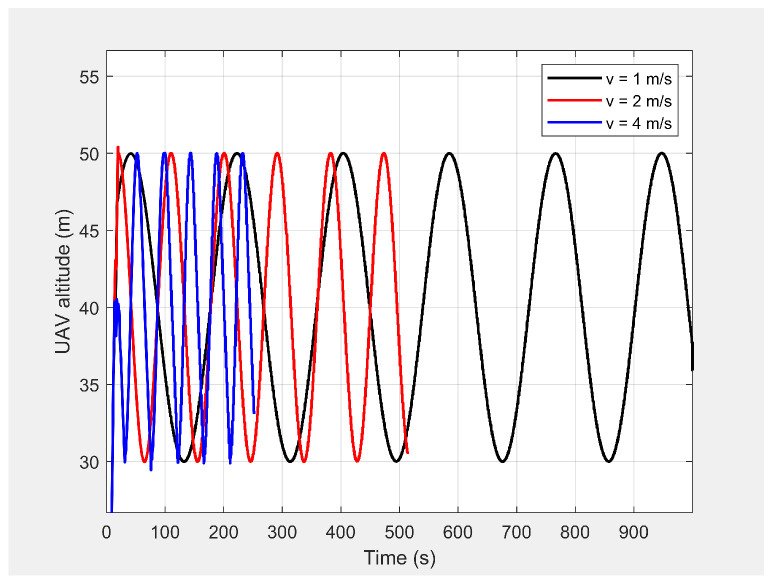
Altitude of the UAV’s planned path when following the MGV while moving at different constant velocities.

**Figure 11:**
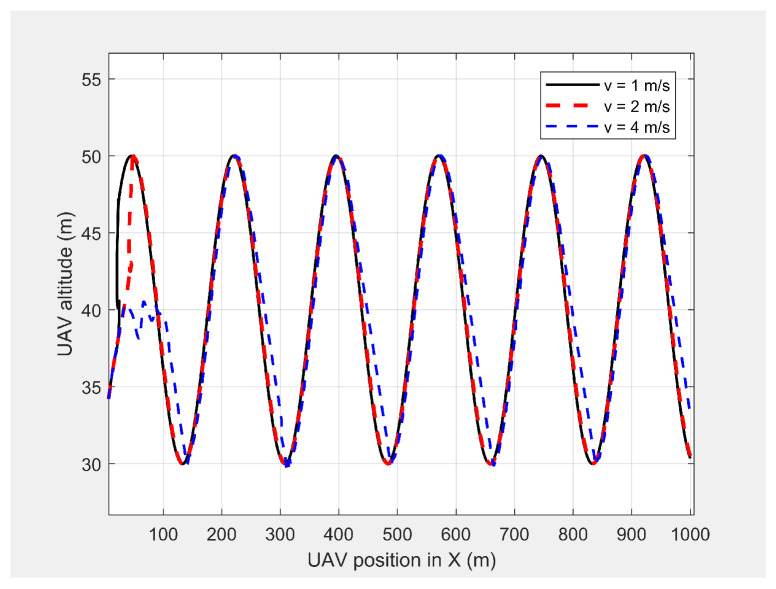
The UAV’s planned path in 2D when following the MGV while moving at different constant velocities.

**Figure 12:**
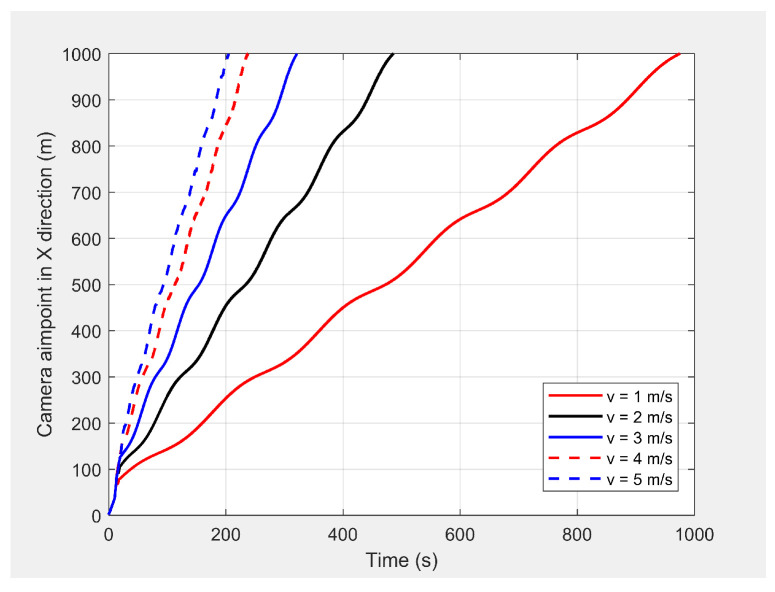
The camera’s aim point for the UAV’s planned path when following the MGV while moving at different constant velocities.

Finally, the MGV’s velocity was set at different constant velocities ranging from 1 m/s to 5 m/s for its 1000 m straight path. The MGV required $\rho_{min}$ and *L* were selected as $142\;{\mathrm{pixel}/m}^{2}$ and 175 m to achieve the sinusoidal amplitude of 10 m for all the selected velocities of the MGV and to clearly show the change of wavelength and frequency for different velocities of the MGV. Figure 9 shows the horizontal velocity of the UAV for different constant velocities for the MGV ranging from 1 m/s to 5 m/s. The UAV reached its maximum velocity in the first few minutes due to the higher relative displacement between the UAV and the MGV. The UAV then maintained a unique horizontal velocity as it followed the MGV. The ED-APF changed the frequency of the path with respect to time while maintaining a constant number of sinusoidal waves with respect to the position, as depicted in Figure 10 and Figure 11, due to the fact that the ED-APF’s vertical attractive force was a function of the UAV’s displacement. The planned path was sinusoidal along the X-axis with a wavelength of 175 m with regard to the MGV’s velocity. However, the UAV’s altitude versus time graph shows the sinusoidal path time periods of 182, 91, and 45.5 s, in line with the MGV’s velocities of 1, 2, and 4 m/s, respectively. Figure 12 shows the camera’s aim point of the UAV’s planned path while following the MGV where the average gradient was greater than 92% around the horizontal velocity of the UAV. The difference between the average gradient and the corresponding velocity decreased with the MGV’s velocity. This was because the amplitude of the sinusoidal path decreased with the MGV’s velocity due to the PX4-autopilot’s limitations.

### 5.5. Performance of the ED-APF for the MGV’s Variable Velocity Motion Profile

**Figure 13:**
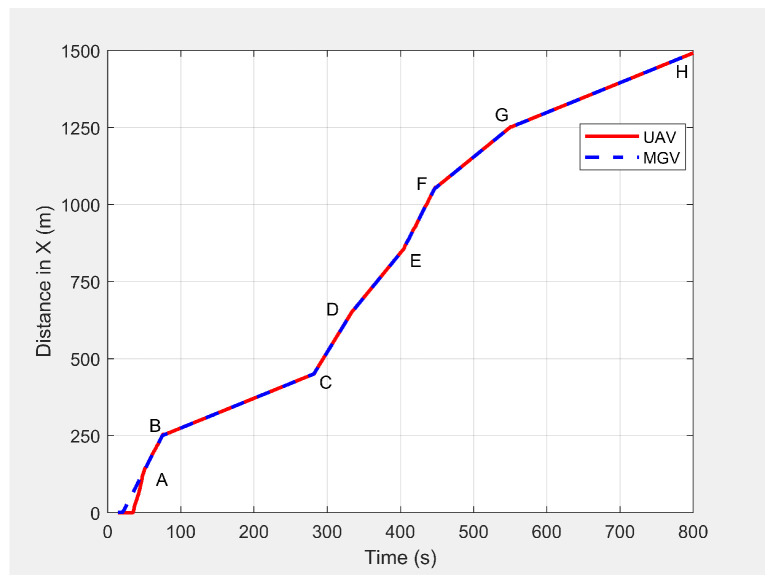
Displacement of the UAV’s planned path when following the MGV while moving at a varying velocity.

**Figure 14:**
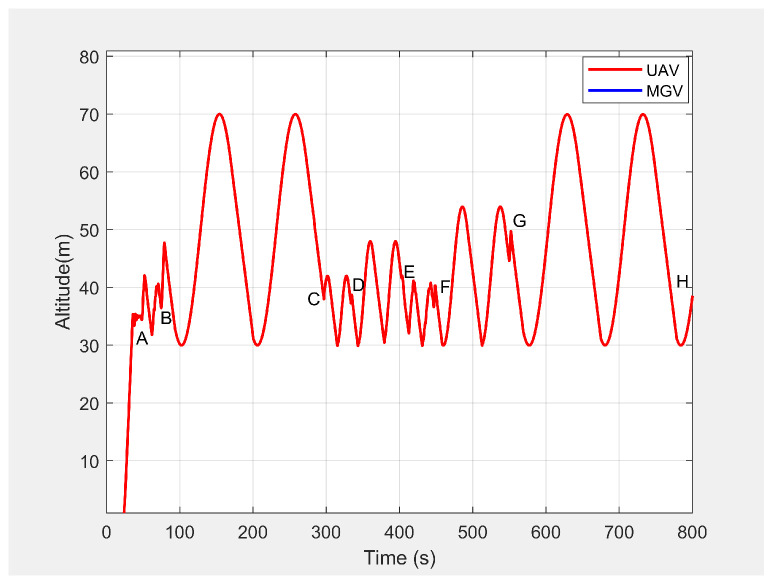
Altitude of the UAV’s planned path when following the MGV while moving at a varying velocity.

**Figure 15:**
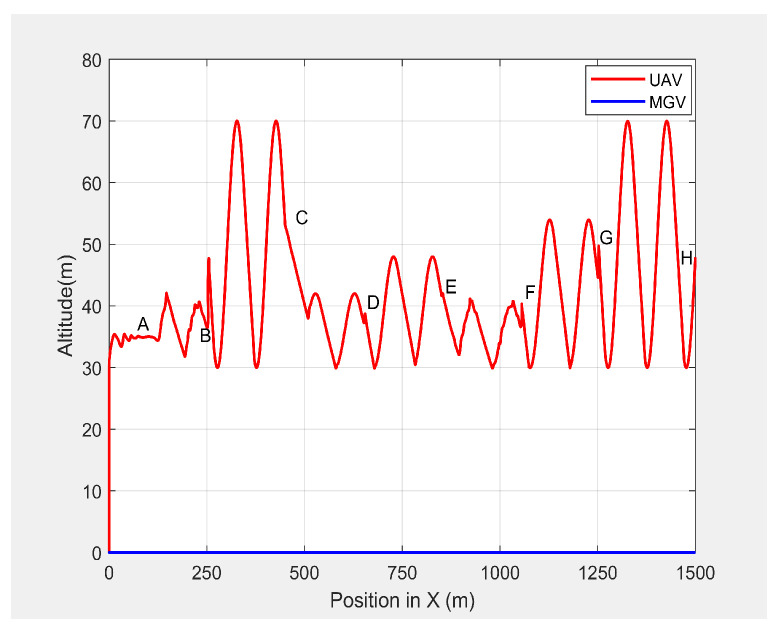
The 2D plot of the UAV’s altitude and position when following the MGV while moving at a varying velocity.

**Figure 16:**
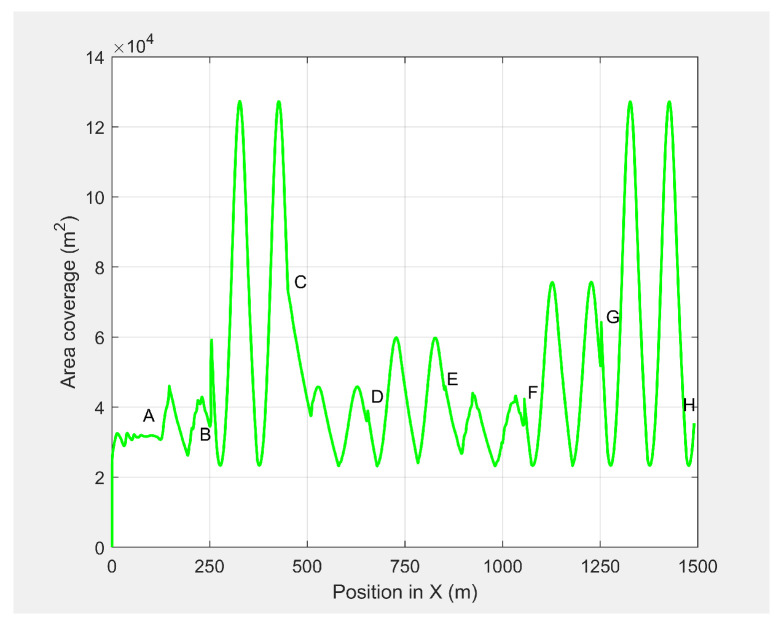
The UAV’s look-ahead coverage when following the MGV while moving at a varying velocity.

In the third set of simulation experiments, the ED-APF’s performance was evaluated as the MGV moved at a varying velocity on a straight path of 1500 m. The MGV’s requested visual quality was chosen as $\rho_{min}=193\;{\mathrm{pixel}/m}^{2}$, which allowed the UAV to achieve a 20 m peak amplitude and to clearly show the change of amplitude and corresponding look-ahead and coverage for different velocities of the MGV. *L* and θ were set to 100 m and $42.35^{\circ }$, respectively. Figure 13 shows the displacement versus time of the UAV following the MGV while moving along the X-direction with a varying velocity in the range of 1 to 5 m/s. The MGV started its horizontal motion at 5 m/s, and the UAV moved vertically until it reached (0,0,10) before it started following the MGV. The UAV followed the MGV along a sinusoidal path while accelerating in the horizontal direction. The UAV flew at a horizontal distance of 140 m from the MGV and followed the MGV for 110 m at 5 m/s to reach Point B. Table 2 summarizes the motion profiles of the MGV and UAV and the look-ahead distance, coverage, and pixel density. Figure 14 shows the altitude of the UAV’s planned path versus time. It shows how the wavelength increased with the amplitude; a larger amplitude indicates a faster velocity of the UAV in the horizontal direction. However, the UAV maintained a wavelength of 100 ± 5 m in the X-direction regardless of the velocity, as shown in Figure 15. As shown in Figure 16, the maximum and minimum look-ahead coverages of 6.22 × $10^{4}$ and 1.14 × $10^{4}$ m${}^{2}$ were achieved at altitudes of 70 and 30 m, respectively.

### 5.6. Performance of the ED-APF for the MGV’s Variable Heading Motion Profile

**Figure 17:**
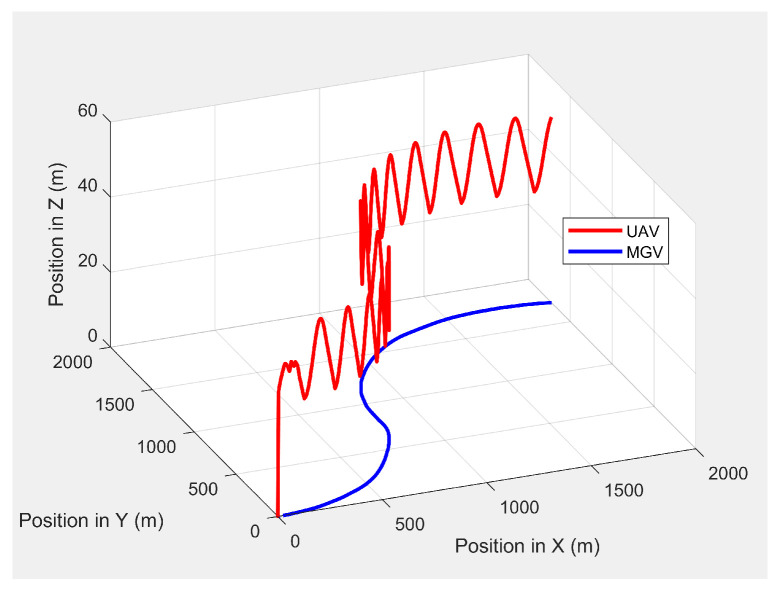
The UAV’s planned path when following the MGV while moving along an unstructured path.

**Figure 18:**
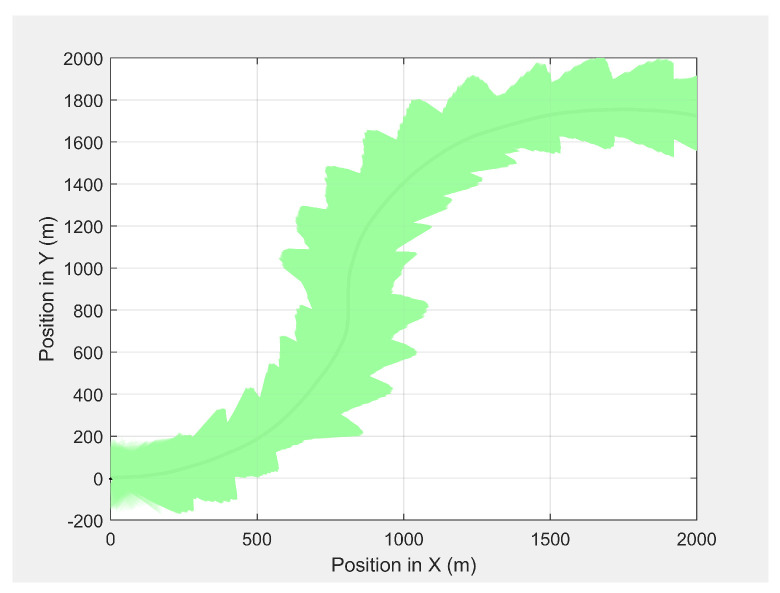
The camera’s footprint as the UAV follows the MGV while moving along an unstructured path.

In the fourth set of simulation experiments, the ED-APF’s performance was evaluated when the MGV was moving along an unstructured path of 3000 m at a speed of 4 m/s. The MGV’s requested visual quality was chosen as $\rho_{min}=\;142\;{\mathrm{pixel}/m}^{2}$, and *L* and θ were set to 175 m and $49.34^{\circ }$, respectively. With the ED-APF, the UAV successfully followed the MGV moving along an unstructured path, as shown in Figure 17. The UAV’s planned path had an altitude of 40 m, oscillating with an amplitude of 10 m. The relative position error had a mean value of 0.06 m and a standard deviation of 0.09 m. Figure 18 shows the camera’s footprint of the UAV. The camera captured a maximum area of $6.5\times 10^{4}\;m^{2}$ and a minimum area of $2.3\times 10^{4}\;m^{2}$ at altitudes of 50 and 30 m, respectively.

### 5.7. The ED-APF’s Performance for Obstacle Avoidance

To evaluate the performance of the ED-APF with regard to obstacle avoidance, two scenarios were considered in the simulation experiments. In both scenarios, the MGV moved in a straight line along the X-direction at varying velocities. In the first scenario, only static obstacles were considered. Two symmetric cylindrical obstacles, each with a radius of 5 m and a height of 40 m, were placed at (120,5.8,0) and (120,−5.8,0) to evaluate the ability of the ED-APF to handle symmetric obstacles along the UAV’s planned path. A bridge-shaped obstacle with a height of 55 m, a width of 40 m, and a length of 30 m was placed at (200,0,0) to evaluate the performance when a large obstacle was on the UAV’s planned path. Another two cylinders, each with a height of 60 m and a radius of 8 m, were placed at (350,9.5,0) and (450,−9.5,0) to evaluate the performance when large obstacles were near the UAV’s planned path. The velocity of the MGV was varied to evaluate its impact on the UAV while in obstacle avoidance mode. The MGV’s required visual quality was chosen as $\rho_{min}=142\;{\mathrm{pixel}/m}^{2}$, and *L* and θ were set to 175 m and $49.34^{\circ }$, respectively.

**Figure 19:**
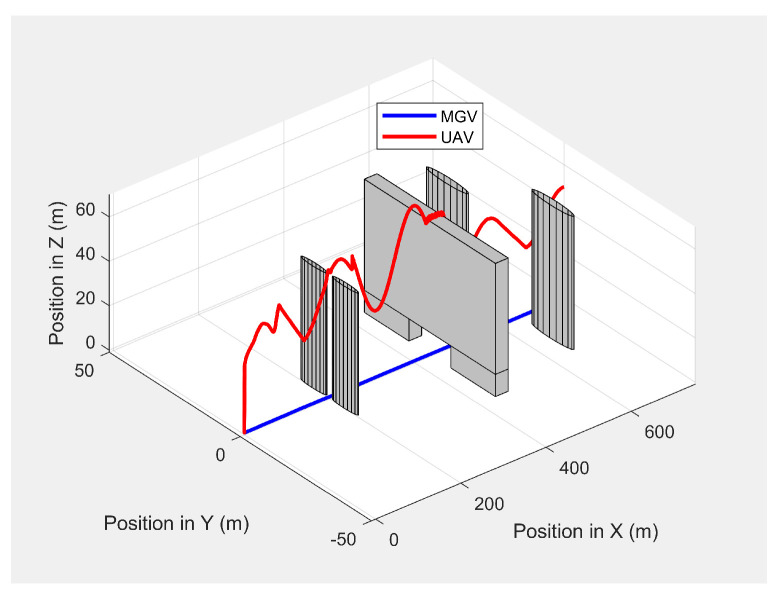
The UAV’s planned path in 3D when following the MGV in the presence of static obstacles.

**Figure 20:**
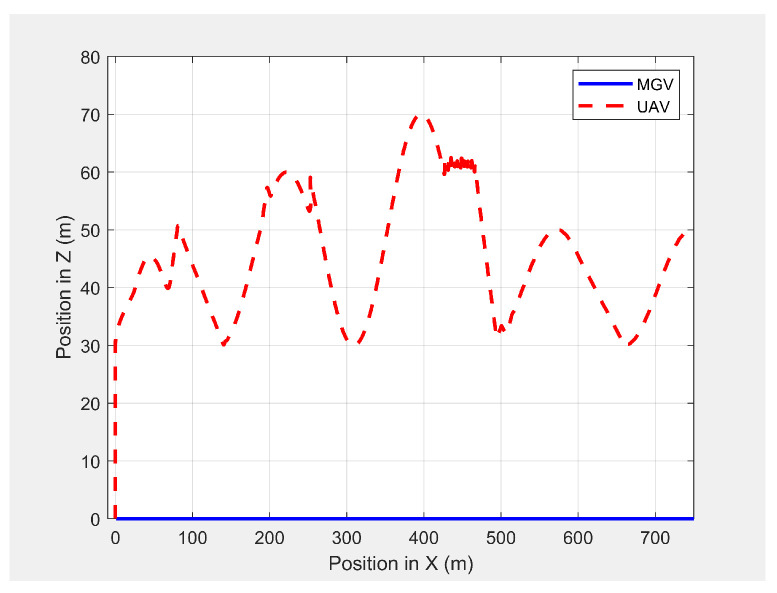
Variation in the altitude of the UAV’s planned path with the horizontal position when following the MGV in the presence of static obstacles.

Figure 19 shows the paths of the MGV and UAV in 3D in the presence of static obstacles and with the MGV’s velocity varying. The UAV successfully followed the MGV along a sinusoidal path by changing its altitude to avoid collisions. The UAV was clearly able to follow the MGV regardless of the obstacles. The UAV completed a full sinusoidal path every 175 m even in the presence of obstacles, as shown in Figure 20.

**Figure 21:**
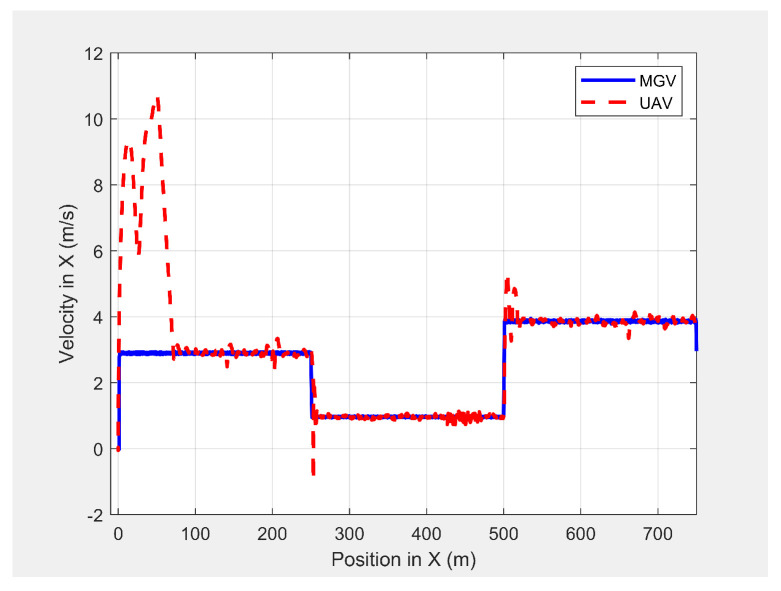
The UAV’s velocity in the horizontal direction when following the MGV while moving at varying velocities in the presence of static obstacles.

**Figure 22:**
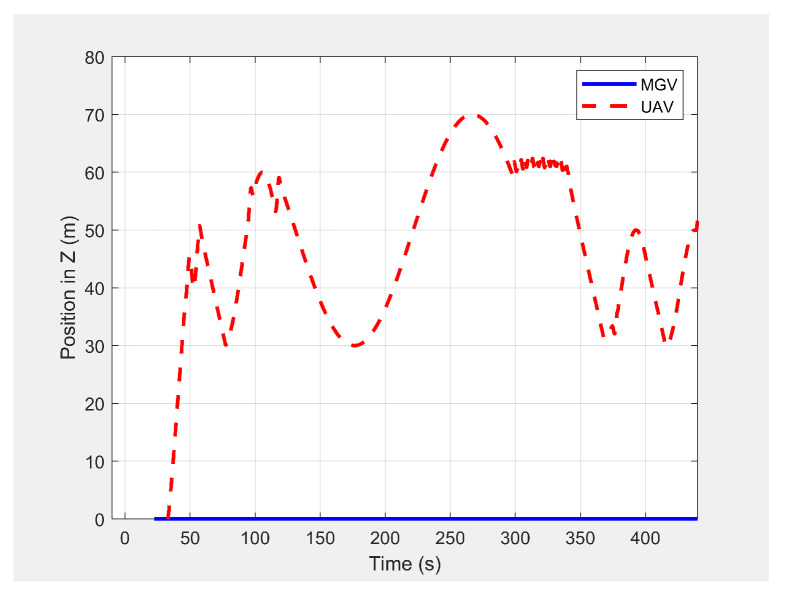
Variation in the altitude of the UAV’s planned path with time when following the MGV while moving at varying velocities in the presence of static obstacles.

Figure 21 shows the velocity profile of the MGV and the horizontal velocity component of the UAV. The UAV had a relative velocity of 0.13 m/s and a standard deviation of 0.2 m/s at the MGV’s velocity of 3 m/s. These changed to 0.11 m/s and 0.15 m/s, respectively, at the MGV’s velocity of 1 m/s, 0.18 m/s, and 0.21 m/s, respectively, and at the MGV’s velocity of 4 m/s. Figure 22 shows how the UAV changed the amplitude and wavelength of its path based on the MGV’s velocity.

**Figure 23:**
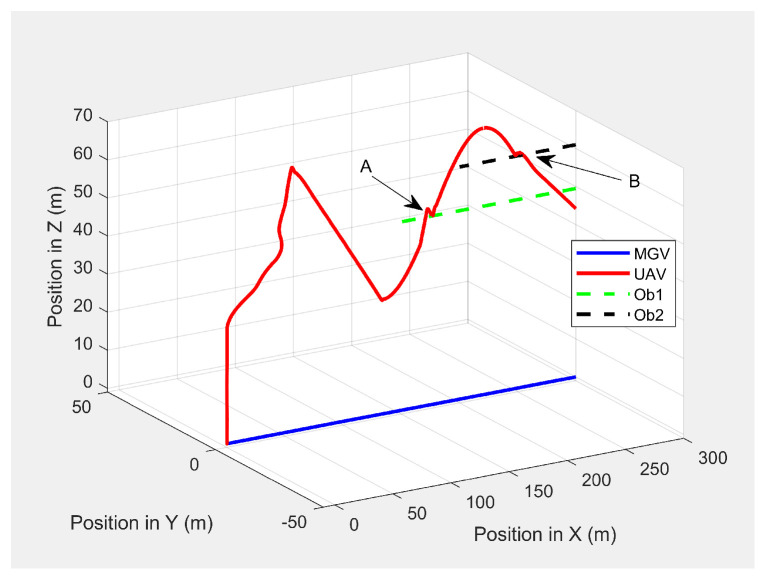
The UAV’s planned path in 3D when following the MGV in the presence of dynamic obstacles.

**Figure 24:**
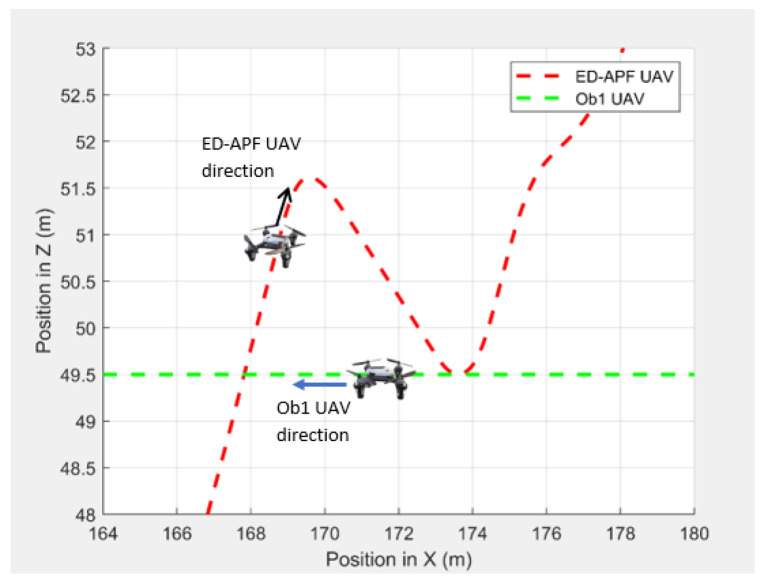
The UAV’s planned path for dynamic obstacle avoidance when encountering another UAV (Ob1).

**Figure 25:**
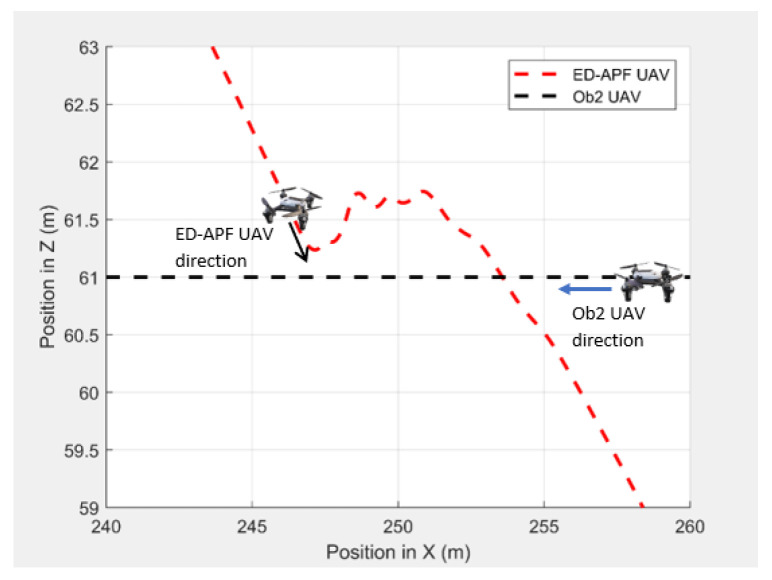
The UAV’s planned path for dynamic obstacle avoidance before encountering another UAV (Ob2).

**Figure 26:**
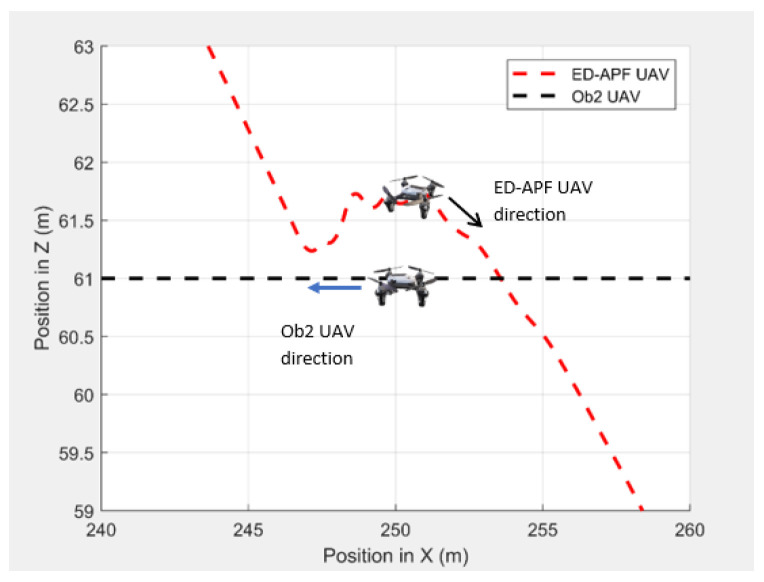
The UAV’s planned path for dynamic obstacle avoidance when encountering another UAV (Ob2).

In the second scenario, two dynamic obstacles were considered: two UAVs moving at 1 m/s along the negative X-direction at altitudes of 49.5 m (Ob1) and 61 m (Ob2). The MGV moved at 2 m/s along the positive X-direction. Figure 23 shows the 3D path of the UAV following the MGV in the presence of dynamic obstacles. The UAV encountered Ob1 and Ob2 at (170,0,49.5) and (250,0,61), respectively. The UAV successfully changed its altitude in the presence of the dynamic obstacles to avoid collision and then continued following the MGV along its sinusoidal path. Figure 24 shows the variation in the altitude of the UAV before and while encountering a dynamic obstacle. Figure 25 and Figure 26 show the variation in the altitude of the UAV in the presence of the dynamic obstacle, which was Ob2, the UAV, before they passed each other and after they passed each other. The UAV successfully changed its altitude for obstacle avoidance while following the MGV.

### 5.8. Comparison of the ED-APF with the General APFs and the D-APF

To compare the performance of the ED-APF against existing APFs, two simulation experiments were conducted. The first experiment evaluated the performances of the ED-APF against existing APFs when the MGV was moving in an open-plain environment. The second experiment evaluated the performances of the ED-APF against existing APFs in an environment with scattered objects where the objects were not obstacles for either the UAV or the MGV, but they acted as occlusions to the UAV’s camera along the MGV’s route. The camera attached to the UAV was set to *L* = 175 m and $\theta =49.34^{\circ }$, and the MGV’s requested visual information quality was $\rho_{min}=100\;{\mathrm{pixel}/m}^{2}$.

#### 5.8.1. Open-Plain Environment

**Figure 27:**
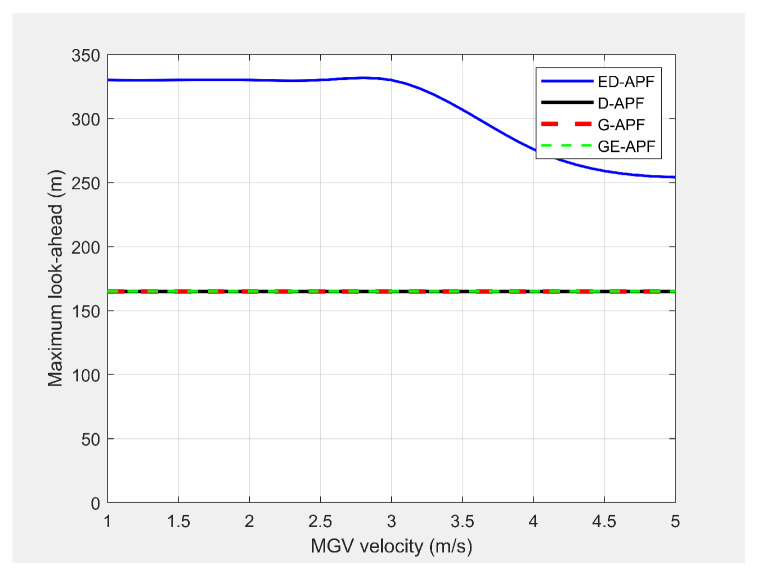
Comparison of the look-ahead distance for different velocities of the MGV.

**Figure 28:**
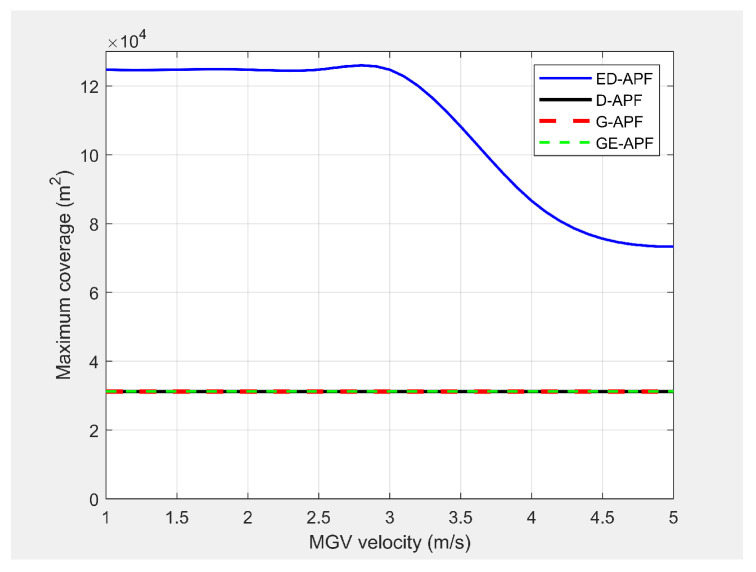
Comparison of the maximum coverage for different velocities of the MGV.

**Figure 29:**
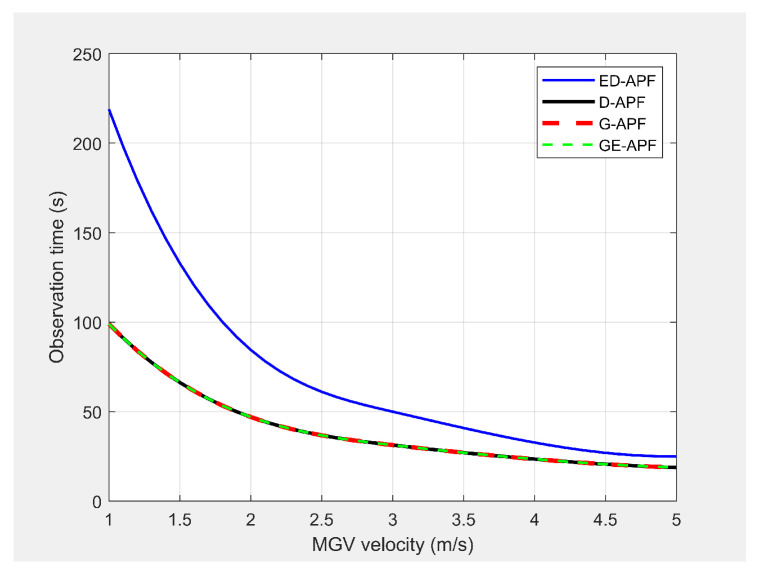
Comparison of the observation time for different velocities of the MGV.

In the first experiment, the MGV moved for 1000 m along the X-direction in an obstacle-free environment with constant velocities from 1 m/s to 5 m/s. Figure 27 and Figure 28 show the simulated look-ahead distance and coverage for the ED-APF and existing APFs. With the general APFs and the D-APF, the UAV achieved a look-ahead coverage of $3.1\times 10^{4}\;m^{2}$ and a look-ahead distance of 165 m for all velocities of the MGV due to the uniform flight altitude of 30 m. The ED-APF had a maximum coverage of $12.3\times 10^{4}\;m^{2}$ and a look-ahead distance of 330 m when the MGV’s velocity was less than 3 m/s. However, the maximum coverage provided by the UAV was then reduced due to the limits of the sinusoidal amplitude. The results showed that the proposed ED-APF provided better look-ahead distance and coverage compared to the general APFs and the D-APF. Besides, it had a better and longer observation time, as shown in Figure 29, of the MGV’s route. This was useful for identification purposes as objects present along the MGV’s route were covered for longer times.

#### 5.8.2. Environment with Visual Occlusions

In the second experiment, the MGV moved for 1500 m along the X-direction at a speed of 3 m/s in an environment with objects acting as occlusions to the UAV’s camera along the MGV’s route. Initially, the MGV passed between two symmetric and cylindrical objects with a radius of 10 m and a height of 12 m positioned at (580360, 1520,0) and (360,−20,0), which did not act as obstacles to either the UAV or the MGV. Later, the MGV moved under a bridge object, with a length of 10 m, a width of 80 m, and a height of 12 m positioned at (880,0,0), which blocked the direct line-of-sight of the UAV’s camera to the MGV’s route. Finally, the MGV moved near by a cylindrical object, with a radius of 10 m and a height of 12 m positioned at (1230,20,0), which partially blocked the line-of-sight of the UAV’s camera to the MGV’s route on the left-hand side.

**Figure 30:**
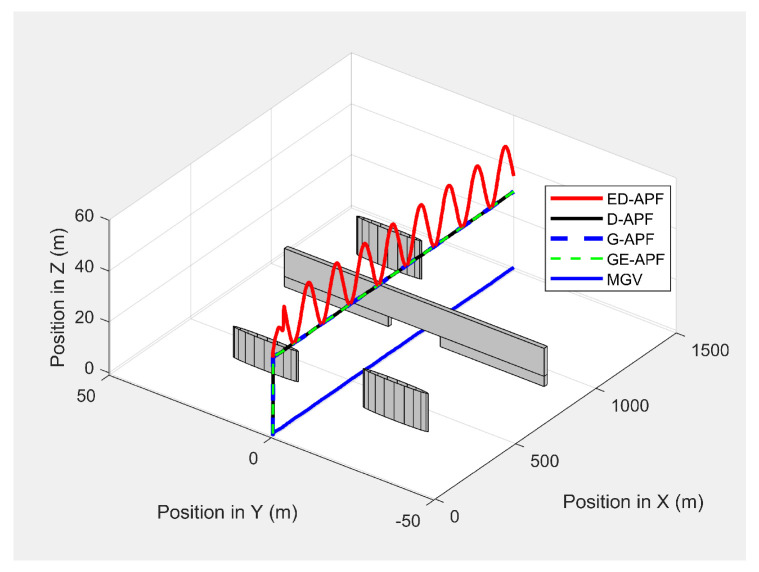
The UAV’s planned path in 3D when following the MGV while moving in an environment with different objects.

**Figure 31:**
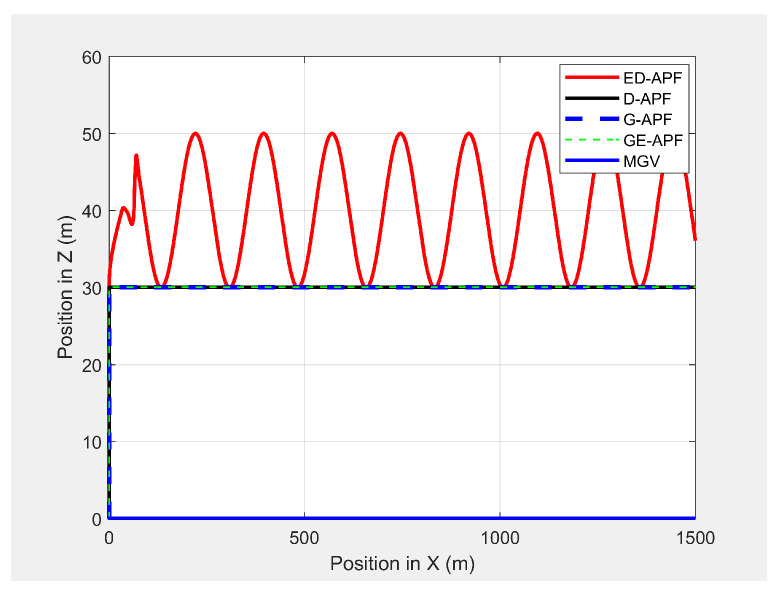
The UAV’s planned path in 2D when following the MGV while moving in an environment with different objects.

**Figure 32:**
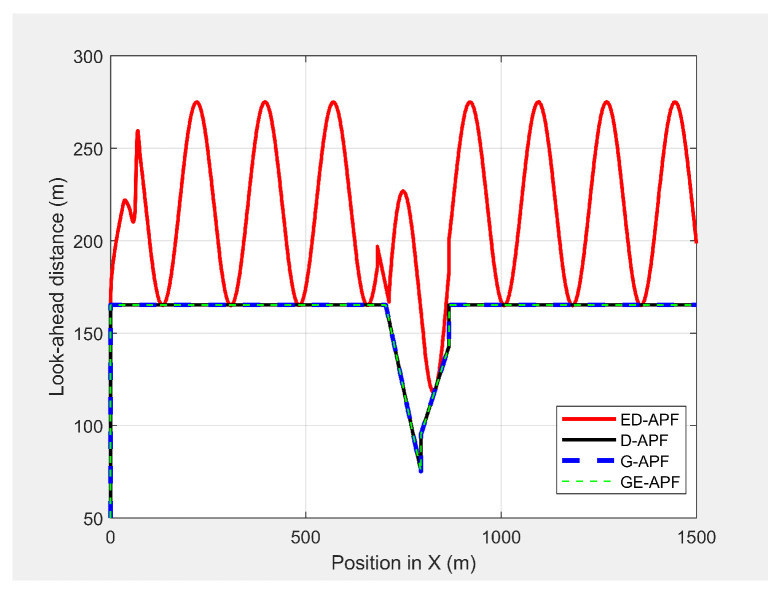
Look-ahead distance along the X-axis vs. the MGV’s position while moving in an environment with different objects.

**Figure 33:**
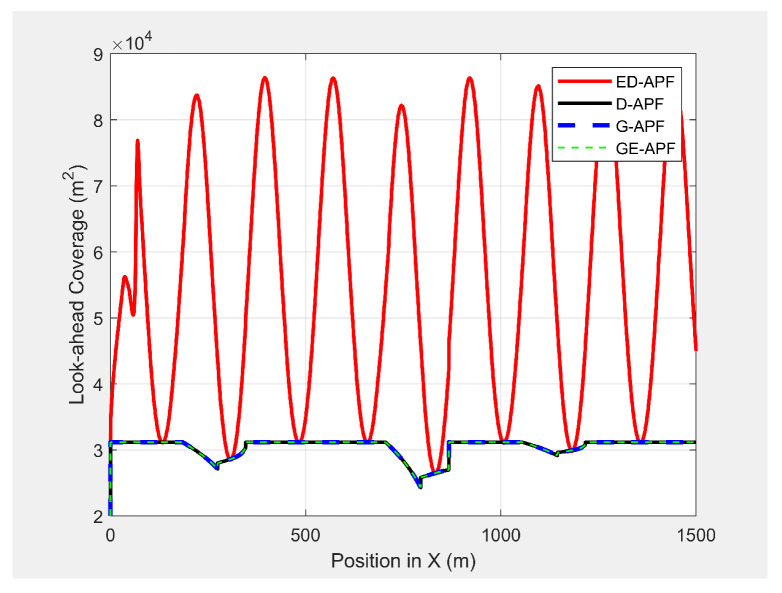
Look-ahead coverage vs. the MGV’s position while moving in an environment with different objects.

As shown in Figure 30, the ED-APF allowed the UAV to follow the MGV along a sinusoidal path while the general APFs and the D-APF maintained the UAV at a constant altitude of 30 m. The UAV spent the first 80 m following the MGV in a stable manner. With the ED-APF, the UAV made a full sinusoidal wave within 175 m with an amplitude of 10 m, as shown in Figure 31, while maintaining an average horizontal velocity of 2.94 ± 0.16 m/s. With the D-APF, G-APF, and GE-APF, the UAV had average velocities of 2.97 ± 0.11, 2.92 ± 0.21, and 2.87 ± 0.22 m/s, respectively. Figure 32 and Figure 33 show the look-ahead distance and coverage. The general APFs and the GE-APF achieved a look-ahead distance of 165 m until the UAV reached the point (705,0,30) and a look-ahead distance of 197 m until the UAV reached the point (683,0,36), respectively, due to facing the bridge object blocking the direct line-of-sight from the UAV’s camera to the MGV’s route. Although, the cylindrical objects did not interrupt the look-ahead distance, the look-ahead coverage decreased due to the blocking of the direct line-of-sight of the UAV’s camera. Therefore, the general APFs and GE-APF achieved a peak look-ahead coverage of $3.1\times 10^{4}\;m^{2}$ until the UAV reached the point (185,0,30) and $8.3\times 10^{4}\;m^{2}$ until the UAV reached the point (225,0,46). Besides, the ED-APF had a maximum coverage of $8.7\times 10^{4}\;m^{2}$ and a look-ahead distance of 275 m after the last object was avoided and the environment was open and obstacle free. Therefore, the proposed ED-APF provided better look-ahead and reconnaissance support compared with the other path planning techniques. This was achieved through the UAV changing its altitude along a sinusoidal path as it followed the MGV.

## 6. Conclusions

In this paper, a novel online 3D path planning technique for multirotor UAVs equipped with nongimbal camera, named the ED-APF, was proposed. This approach supports the reconnaissance and exploration of areas and routes ahead and beyond the MGVs’ sensor range by formulating the path planning goal as a combination of follow and coverage problems. The ED-APF’s proposed vertical component of the attractive force guided the UAV to follow the MGV in a vertical sinusoidal path with a constant wavelength, simultaneously alternating between broad and precise coverages. The wavelength of the sinusoidal path was calculated as a function of the camera field-of-view and the gimbal angle. The flying altitude was calculated as a function of the pixel density requirement of the MGV and the speed of the MGV. The ED-APF’s repulsive force was utilized to avoid static and dynamic obstacles that could collide with the UAV.

The performance of the proposed ED-APF path planning technique was validated in different realistic simulation scenarios, including the MGV moving along structured (i.e., straight line) and unstructured (i.e., uneven) paths with constant and varying velocities. The ED-APF showed average relative displacement errors of 1.0%, 1.2%, and 1.5% per meter along the horizontal direction of motion and standard deviations of 0.08, 0.12, and 0.15 m for velocities of the MGV of 1.0, 3.0, and 5.0 m/s, respectively. Besides, the ED-APF technique had the capability to maintain the average relative displacement error within less than 2% per meter along the vertical direction. Cylinder- and bridge-shaped obstacles were used to evaluate the performance of the ED-APF in the presence of static obstacles. The simulation results confirmed that the UAV could successfully avoid static obstacles while following the MGV. Besides, the UAV could also change its altitude to avoid collisions with dynamic obstacles.

The simulation results confirmed that the proposed ED-APF technique could expand the look-ahead coverage by 445%, 156%, and 78% at velocities of the MGV of 1, 3, and 5 m/s, respectively, for a 42.35${}^{{}^{\circ}}$ gimbal angle, a 100 m sinusoidal wavelength, and a 94${}^{{}^{\circ}}$ camera FoV. The corresponding look-ahead distance increased by 133%, 60%, and 33% at of velocities of the MGV of 1, 3, and 5 m/s, respectively. Besides, the changing *L* and θ studies showed that increasing θ required increasing *L*; therefore, the ED-APF approach could increase the look-ahead and aerial coverage by 160% and 478% at a velocity of the MGV of 4 m/s and *L* of 200 m for a visual quality of $\rho_{min}=58\;{\mathrm{pixel}/m}^{2}$.

The proposed ED-APF approach was also compared against the D-APF technique and the general APF techniques in scenarios where the flying environment had objects that were not obstacles for the UAV. The ED-APF showed superiority to the D-APF and the general APF techniques in terms of improving the look-ahead distance and coverage. This was mainly achieved by the change of the UAV’s altitude in a vertical sinusoidal path, which minimized the blockage of the direct line-of-sight by the obstacles. Therefore, the ED-APF was more efficient than other existing path planning techniques for multirotor UAVs in providing reconnaissance and look-ahead support for MGVs.

## Figures and Tables

**Table 1:** The ED-APF’s look-ahead distance and coverage for different $L_{1}$ values at the MGV’s velocity of 4 m/s.

ED-APF Parameters		MGV Minimum Sensing	Look-Ahead Distance (m)		Look-Ahead Coverage (×10^4^ m^2^)	
$L_{1}$ **(m)**	θ **(Degrees)**	**Distance (m)**	**Max**	**Min**	**Max**	**Min**
94	42.35	6.05	131.2	96.0	2.24	1.14
141	47.74	9.05	225.6	141.1	5.96	2.35
165	49.34	9.06	275.6	165.6	8.73	3.21
190	50.52	10.66	341.0	190.1	12.94	4.00
239	52.21	11.67	445.2	240.1	21.23	6.13

**Table 2:** Look-ahead distance and coverage for different velocities of the MGV.

Motion Points	Average Velocity in the Horizontal Direction (m/s)		Look-Ahead Coverage (10^4^ m^2^)		Look-Ahead Distance (m)		Pixel Density (pixel/m^2^)	
	**MGV**	**UAV**	**Max**	**Min**	**Max**	**Min**	**Max**	**Min**
A-B	4.98 ± 0.03	4.97 ± 0.04	2.03	1.14	125	94	1051	591
B-C	0.99 ± 0.01	0.99 ± 0.02	6.22	1.14	219	94	1051	193
C-D	4.00 ± 0.03	3.99 ± 0.04	2.24	1.14	131	94	1051	536
D-E	2.99 ± 0.02	2.98 ± 0.03	2.92	1.14	150	94	1051	410
E-F	4.98 ± 0.03	4.98 ± 0.04	2.03	1.14	125	94	1051	591
F-G	2.00 ± 0.02	2.00 ± 0.03	2.70	1.14	169	94	1051	324
G-H	0.99 ± 0.01	0.99 ± 0.02	6.22	1.14	219	94	1051	193

## Data Availability

Data of our study are available upon request.
